# The Association of Health-Related Factors with Leisure-Time Physical Activity among Adults with COPD: A Cross-Sectional Analysis

**DOI:** 10.3390/healthcare10020249

**Published:** 2022-01-28

**Authors:** Mei-Lan Chen, Li-Sheng Chen, Yen Tzu Chen, Douglas S. Gardenhire

**Affiliations:** 1 School of Nursing, Georgia State University, Atlanta, GA 30303, USA; 2 Department of Respiratory Therapy, Georgia State University, Atlanta, GA 30303, USA; lchen48@student.gsu.edu (L.-S.C.); dgardenhire@gsu.edu (D.S.G.); 3 Department of Physical Medicine and Rehabilitation, University of Michigan, Ann Arbor, MI 48105, USA; yentchen@med.umich.edu

**Keywords:** chronic obstructive pulmonary disease, COPD, physical activity, health attitudes, health appraisals, negative affect, positive affect

## Abstract

This study aimed to examine the association of health attitudes, health appraisals and affective experience to leisure-time physical activity in adults with chronic obstructive pulmonary disease (COPD). Cross-sectional analyses were conducted with a sample of 274 adults with COPD drawn from the second wave of the Midlife in the United States (MIDUS 2) Study. Chi-square analyses and independent *t*-tests were used to test the differences between physically active and inactive COPD patients (active group versus inactive group) for all study variables. Multiple logistic regression was used to examine the association of each study variable with leisure-time physical activity. The results showed that there were significant differences between the active and inactive groups in terms of age, education, functional limitations, health attitudes, health appraisals and affective experience. After controlling for socio-demographic variables and functional limitations, beliefs about the importance of physical fitness and strength for a good life and comparative health appraisals were significantly related to physical activity. However, neither negative nor positive affect was associated with physical activity status. Modifiable factors, such as health attitudes toward physical fitness and strength, as well as health appraisals, should be considered for developing effective physical activity promotion interventions among COPD patients.

## 1. Introduction

Chronic lower respiratory disease, primarily chronic obstructive pulmonary disease (COPD), was the 4th leading cause of death in the United States [1]. An estimated 15.5 million adults in the United States had been diagnosed with COPD [2]. COPD is characterized by a progressive deterioration in pulmonary function, and is associated with various symptoms such as dyspnea, fatigue, sputum production, wheezing and chest tightness. Symptoms of COPD may contribute to an increase in anxiety, depression and the risk of exacerbation and worse prognosis in the COPD population [3]. The burden of COPD on patients with the disease includes high healthcare cost, decreased quality of life, impaired daily functioning, poor quality of sleep, and reduced level of physical activity [3,4].

Compared to healthy adults, engaging in regular physical activity is more difficult for patients with COPD due to their symptoms (e.g., fatigue, feeling of breathlessness, muscle deconditioning) [3,5,6]. Additionally, evidence shows that COPD patients are more likely to have activity limitations and mobility impairment compared to people without COPD [7]. Being physically active may alleviate burdens or delay impairment due to COPD. Research has shown that patients with COPD who participated in physical activity were less likely to report COPD-related hospital admissions and mortality than those who did not [8]. In contrast, COPD patients who had lower levels of physical activity were more likely to report having chronic anxiety, chronic lumbar back pain, urinary incontinence, cataracts and chronic constipation than their counterparts [9]. Although there is strong evidence showing the health benefits of physical activity, participation remains low among COPD patients [5,6,10]. Thus, it is important to identify the factors that are related to physical activity participation among the COPD population.

Previous research demonstrated that some factors were associated with physical activity in COPD patients, including age, sex, race/ethnicity, education, marital status, smoking, obesity and functional limitations [11,12,13,14,15,16,17]. Several studies focused on the general healthy population found that psychosocial factors were related to physical activity status [18,19,20,21]. However, limited studies have examined psychosocial variables in relation to physical activity engagement among COPD patients. Factors influencing leisure-time physical activity remain unclear in this population, given their health conditions and vulnerable characteristics. In addition, several health-related factors, such as health attitudes, health appraisals, as well as positive and negative affect, have not yet been examined in relation to physical activity participation in COPD patients. Thus, the purpose of this study was to examine the association of health attitudes, health appraisals, and affective experience to leisure-time physical activity among adults with COPD.

## 2. Materials and Methods

### 2.1. Data Source and Study Design

The current study was conducted as a secondary analysis of data from the second wave of the Midlife in the United States (MIDUS 2) study, a 2004–2006 national survey in the United States [21]. The data from MIDUS 2, which is publicly available, were obtained from the Inter-University Consortium for Political and Social Research (ICPSR) [21]. A detailed description of MIDUS 2 has been previously reported by Ryff and colleagues [21], and is available at http://www.midus.wisc.edu (accessed on 21 March 2020).

In the current study, MIDUS 2 respondents who have experienced, been diagnosed with, or treated for COPD were selected as subjects.

### 2.2. Measurements

#### 2.2.1. Socio-Demographic Characteristics and Functional Limitations

Socio-demographic characteristics and functional limitations were selected as covariates based on the previous literature [11,12,13,14,15,16,17]. Socio-demographic characteristics included age (≤60 years old vs. >60 years old), sex (female vs. male), race/ethnicity (White vs. Black and other), education (high school or below vs. some college, college graduate, or higher education), marital status (not married vs. married), smoking status (non-smokers vs. smokers) and weight status (not obese vs. obese). A body mass index (BMI) of 30 kg/m^2^ or greater was categorized as obese. To evaluate functional limitations, participants were asked to report “how much their health limited: lifting or carrying groceries; bathing or dressing; climbing several flights of stairs; climbing one flight of stairs; bending, kneeling or stooping; walking more than a mile; walking several blocks; walking one block” [21]. All items were rated on a scale ranging from 1 (a lot) to 4 (not at all). Higher scores indicated less functional limitation. Cronbach’s α for the current study was 0.94.

#### 2.2.2. Leisure-Time Physical Activity

To assess participation in physical activity, participants were asked to report the frequency of their engagement in both moderate and vigorous physical activity during their leisure or free time in the summer and winter [21]. In the current study, responses for the frequency of physical activity were categorized according to whether respondents met physical activity recommendations based on the 2018 Physical Activity Guidelines Advisory Committee Scientific Report [22] and previous studies [19,23,24]. Participants who reported never, less than once a month, or once a month for both moderate and vigorous physical activity during either summer or winter were classified as physically inactive. Participants who reported several times per week or more for either moderate or vigorous physical activity during both summer and winter were classified as physically active [19,23,24].

#### 2.2.3. Health Attitudes toward Fitness and Strength

To evaluate health attitudes toward fitness and strength, participants were asked to report whether they felt “physical fitness and strength are the most important for living a good life” [21].

#### 2.2.4. Health Appraisals

Participants were asked to evaluate themselves today compared to five years ago on four items: energy level, physical fitness, physique/figure and weight [21]. The four items were scored on a scale from 1 (improved a lot) to 5 (become a lot worse). All items were reverse-scored and averaged to assess their health compared to 5 years ago. In the current study, Cronbach’s α was 0.88.

#### 2.2.5. Negative Affect

Negative affect was evaluated in participant responses to six items referring to the question: “During the past 30 days, how much of the time did you feel…” [21]. Items included: so sad nothing could cheer you up, nervous, restless or fidgety, hopeless, everything was an effort and worthless. All items were rated on a scale ranging from 1 (all the time) to 5 (none of the time). These items were reverse scored. A total score was computed by adding the six items; scores could range from 6 to 30. Lower scores indicated fewer negative feelings [25]. In the present study, Cronbach’s α was 0.89.

#### 2.2.6. Positive Affect

Positive affect was assessed in participant responses to six items referring to the question: “During the past 30 days, how much of the time did you feel…” [21]. Items included: cheerful, in good spirits, extremely happy, calm and peaceful, satisfied, and full of life. Responses were on a scale from 1 (all the time) to 5 (none of the time). These items were reverse scored. A total score was computed by adding the six items; scores could range from 6 to 30, with lower scores indicating fewer positive feelings [25]. Cronbach’s α for the present study was 0.91.

### 2.3. Ethical Approval

This study was approved by the Institutional Review Board (IRB) of Georgia State University (IRB reference number: H20653).

### 2.4. Statistical Analysis

Chi-square analyses and independent *t*-tests were used to examine if there were differences between physically active and inactive COPD patients in terms of socio-demographics, functional limitations, health attitudes, health appraisals, as well as positive and negative affect. A multiple logistic regression, adjusting for socio-demographic variables (age, sex, race/ethnicity, education, marital status, smoking status and weight status) and functional limitations, was conducted to examine the association of each study variable with leisure-time physical activity status. Assumptions for logistic regressions, including multicollinearity, were checked prior to statistical analysis, and suggested that there were no violations. In addition, several interactions between socio-demographic variables were tested in the regression models. Model fit was assessed by examining estimated versus observed outcomes in physical activity status using the Hosmer–Lemeshow goodness-of-fit test. The data were analyzed using SPSS version 24 (IBM, Armonk, NY, USA). The significance level was set at *p* < 0.05.

## 3. Results

### 3.1. Characteristics of the Sample

A total of 274 adults with COPD were included in this study. Participants ranged in age from 33 to 81 (mean age 57.32 years ± 12.59). Of the total sample, 30.7% of participants were classified as physically active. In terms of sex, 66.4% self-reported as female and 33.6% self-reported as male. Approximately 89.3% were White. The majority of participants had received education beyond high school (61.3%), were married (58.4%), non-smokers, (81.4%) and not obese (57.7%).

### 3.2. Comparison of Physical Activity Status

Table 1 indicates the results of physical activity status comparisons for all study variables. As shown in Table 1, there were significant differences between the active and inactive groups in terms of age, education, functional limitations, health attitudes, health appraisals and affective experience. Compared to the older age group (>60 years old), the younger age group (≤60 years old) was more likely to engage in regular physical activity. Participants who had a higher level of education were more likely than their counterparts to meet the physical activity guideline. COPD patients with fewer functional limitations were more likely to be active. Compared to COPD patients who were physically inactive, those who engaged in regular physical activity were more likely to indicate that physical fitness and strength are important for a good life. Physically active COPD patients were more likely to report higher ratings on comparisons of four aspects of health (energy, physical fitness, physique/figure and weight) compared to five years ago than their counterparts. Furthermore, COPD patients who reported lower negative affect scores and higher positive affect scores were more likely than their counterparts to meet the physical activity guideline.

### 3.3. Association of Physical Activity Status with Health-Related Factors

The results of multiple logistic regression analyses are presented in Table 2. Belief that physical fitness and strength are important for a good life (OR = 2.47, 95% CI = 1.16–5.24) and comparative health appraisal (OR = 1.90, 95% CI = 1.34–2.71) were positively associated with active status, after adjusting for socio-demographic variables and functional limitations. However, neither negative nor positive affect was associated with physical activity status. No significant interactions were found between socio-demographic variables. The regression model showed a good fit to the data as the Hosmer–Lemeshow test was not significant [X^2^ (8) = 11.89, *p* = 0.156].

## 4. Discussion

COPD patients engage in less physical activity than their healthy counterparts or patients with other chronic diseases [1,5,6,14]. Yet factors influencing physical activity in individuals with COPD remain controversial [10]. The current study investigated the relation of health-related factors to physical activity in a nationally representative COPD population. To the best of our knowledge, this is the first study to examine whether health attitudes, health appraisals and affective experience are associated with leisure-time physical activity participation in adults with COPD. The findings of this study revealed that there were statistically significant differences in age, education level, functional limitations, health attitudes, health appraisals and affective experience between physically active and inactive COPD patients. The results of the present study also indicated that health attitudes and health appraisals were significant predictors of leisure-time physical activity engagement among patients with COPD, after adjusting for socio-demographic factors and functional limitations.

The existing literature suggests that age, education, smoking behavior and functional limitations may impact physical activity levels [5,11,12,13,14,15,26,27]. Consistent with previous research [5,12,13,14,26,27], results from this study indicated that COPD patients with younger age, higher education and fewer functional limitations were more likely than their counterparts to meet physical activity recommendations. However, in contrast to prior research [11,15], there were no statistically significant differences in physical activity status between current smokers and non-smokers. This may be explained by the greatly uneven distribution of smokers and non-smokers in this study population. In addition, pack-years of smoking were not quantified in the MIDUS 2 study, hindering the contribution of tobacco dosage to physical activity in the study participants.

Previous studies suggested that a stronger belief in the importance of physical fitness and strength for a good life, greater ratings of comparative health appraisals, higher levels of positive affect and lower levels of negative affect were associated with a higher level of leisure-time physical activity among middle-aged and older women [19,28]. Similarly, in the current study, physically active COPD patients were more likely to believe physical fitness and strength are most important for living a good life and to report better health when compared to the past than inactive COPD patients. As for affective experience, active participants were more likely to report lower negative affect scores and higher positive affect scores when compared to their counterparts.

In this study, the key findings from a multiple logistic regression analysis indicated that the belief that physical fitness and strength are important for a good life and comparative health appraisals significantly predicted leisure-time physical activity participation in adults with COPD, after controlling for socio-demographic variables and functional limitations. The present results showed that health attitudes toward fitness and strength were positively related to physical activity engagement. Consistent with previous research, findings indicated that COPD participants who considered maintaining fitness and strength important were more likely to stay physically active than their counterparts [29]. Additionally, the result of the current study revealed that comparative health appraisal was positively associated with active status. COPD patients who reported higher ratings of health appraisal scores were more likely to engage in leisure-time physical activity. There is consistent evidence indicating that individuals who had better appraisals of their health compared to their past health were more likely to meet the physical activity guideline when compared to their counterparts [5,6,26,29,30,31].

In contrast with prior studies [5,6,26,31], the results of this study showed that positive affect and negative affect did not significantly predict leisure-time physical activity among adults with COPD, after adjusting for socio-demographic factors and functional limitations. A possible explanation is that since the MIDUS 2 population consisted of twins and siblings, they might have acted as external regulators of COPD patients’ participation in physical activity. This may have minimized the effect of intrinsic affect on physical activity status. Another possible explanation is that the study participants were accustomed to the burdens of COPD and had received enough knowledge to know the importance of physical activity to their health, resulting in the independence of physical activity from daily affect.

Physical activity is a crucial component in maintaining a healthy lifestyle and better quality of life, mitigating the burden of symptoms and healthcare costs and improving physical and mental health in COPD patients [8,9,32,33,34,35,36,37]. Yet physical activity participation in this population remains low [10,32,38,39,40,41,42,43,44]. Obviously, promoting physical activity is essential for adults with COPD. Findings from this study and previous research suggested that health appraisals and health attitudes toward fitness and strength significantly predicted physical activity engagement [5,6,26,29,30,31]. Hence, it is crucial to provide health education on self-ratings and comparisons of health, the health benefits of physical activity and the importance of physical fitness and strength for a good life in order to enhance COPD patients’ physical activity participation [45]. In addition, previous research indicated that peer and family support, social networks, behavioral change strategies and technology-based interventions had positive effects in physical activity promotion [34,35,38,40,41,43,46,47,48]. Studies also showed that functional capacity, self-efficacy and depression and anxiety status were significantly related to physical activity engagement [49,50,51,52]. Moreover, exacerbations of COPD and the burden of symptoms in COPD can influence physical activity levels [1,5,6,14,27,29,42,43,53]. Hence, the combination of symptom self-management education, pulmonary rehabilitation and physical activity intervention is highly recommended for COPD patients. Overall, in order to deliver effective and sustained physical activity interventions for COPD patients, health attitudes, health appraisals, as well as physical and psychosocial factors should be considered for future research and clinical practice.

This study has several limitations. First, since this study is a cross-sectional study, causal relations may not be inferred. Second, the source of the data came from subjectively answered questionnaires in the MIDUS 2 study, introducing a risk of recall bias among participants. In addition, only one item was used to measure health attitudes. Moreover, the associations between study variables might be explained by one or more unmeasured confounding variables (e.g., comorbidities). Additionally, the sample in the active physical activity group in the analyses was relatively small, leading to the low power of the present study. The statistical significance might be lost in the regression model, particularly in the associations of positive affect and negative affect to physical activity status, possibly due to a reduction in statistical power. Furthermore, because the data were not collected recently, considering the pandemic, the perceptions of life and well-being in COPD patients might now be different when compared to the sample used in this study. Therefore, caution is needed in generalizing these findings. Lastly, most participants were White, women, non-smokers and had received higher education, which may contribute to limit the generalizability of the study results.

## 5. Conclusions

Despite evidence showing that physical activity is a vital intervention in maintaining health, many COPD patients do not participate in physical activity. How to improve COPD patients’ physical activity participation remains a challenge. Hence, identifying factors facilitating physical activity in this population is critically important. This study showed that there were significant differences between physically active and inactive COPD patients in terms of age, education, functional limitations, health appraisals, health attitudes toward fitness and strength, positive affect and negative affect. The findings of this study suggest that health attitudes and health appraisals were significant predictors of staying physically active among adults with COPD, after adjusting for socio-demographic factors and functional limitations. Healthcare providers should aim to promote the belief that physical fitness and strength are important for living a good life, along with health comparisons to the past when encouraging COPD patients to engage in leisure-time physical activity.

## Figures and Tables

**Table 1:** Sample characteristics and comparison of physical activity status on all study variables in COPD patients.

Variables	Physical Activity Status		X^2^ or *t*-Test	*p*-Value
	Inactive (n = 178)	Active (n = 79)		
Age (years; mean ± SD)	58.2 ± 12.4	53.5 ± 11.7	t = 2.86	0.005 **
Age (%)			X^2^ = 6.93	0.008 **
≤60 years old	53.4	70.9		
>60 years old	46.6	29.1		
Sex (%)			X^2^ = 0.01	0.942
Female	66.3	65.8		
Male	33.7	34.2		
Race/Ethnicity (%)			X^2^ = 2.89	0.089
White	87.6	94.7		
Black and others	12.4	5.3		
Education (%)			X^2^ = 6.45	0.011 *
High school or below	43.3	26.6		
Some college, college graduate, or higher	56.7	73.4		
Marital status (%)			X^2^ = 0.05	0.828
Not married	40.7	39.2		
Married	59.3	60.8		
Smoking status (%)			X^2^ = 3.63	0.057
Non-smokers	78.7	88.6		
Smokers	21.3	11.4		
Weight status (%)			X^2^ = 0.09	0.761
Not obese	59.0	57.0		
Obese	41.0	43.0		
Functional limitations (mean ± SD)	23.19 ± 7.44	26.76 ± 7.09	t = −3.57	<0.001 ***
Health attitudes toward fitness and strength (%)			X^2^ = 6.30	0.012 *
Not important	80.3	65.8		
Important	19.7	34.2		
Health appraisals (mean ± SD)	2.42 ± 0.84	3.08 ± 1.11	t = −5.21	<0.001 ***
Negative affect (mean ± SD)	10.59 ± 4.76	9.35 ± 3.38	t = 2.06	0.040 *
Positive affect (mean ± SD)	18.69 ± 4.69	20.29 ± 4.44	t = −2.56	0.011 *

Note: * *p* < 0.05, ** *p* < 0.01, *** *p* < 0.001 (two-sided).

**Table 2:** Multiple logistic regression analysis examining variables associated with physical activity status among adults with COPD, controlling for socio-demographic factors and functional limitations (n = 274).

Variables	OR (95% CI)	*p*-Value
Age (≤60 years old ^a^ vs. >60 years old)	0.56 (0.26, 1.19)	0.130
Sex (female ^a^ vs. male)	1.08 (0.53, 2.18)	0.842
Race/ethnicity (White ^a^ vs. Black and other)	0.26 (0.07, 0.99)	0.048 *
Education (high school or below ^a^ vs. some college, college graduate, or higher)	1.36 (0.66, 2.80)	0.410
Marital status (not married ^a^ vs. married)	1.06 (0.54, 2.06)	0.871
Smoking status (non-smokers ^a^ vs. smokers)	0.41 (0.15, 1.16)	0.093
Weight status (not obese ^a^ vs. obese)	1.73 (0.86, 3.51)	0.127
Functional limitations	1.07 (1.00, 1.14)	0.039 *
Health attitudes toward fitness and strength (%) (not important ^a^ vs. important)	2.47 (1.16, 5.24)	0.019 *
Health appraisals	1.90 (1.34, 2.71)	<0.001 ***
Negative affect	1.05 (0.92, 1.20)	0.460
Positive affect	1.05 (0.94, 1.17)	0.427

Note: ^a^ Reference category. CI = confidence interval. The comparison group was those who did not meet physical activity guidelines (inactive status). * *p* < 0.05, *** *p* < 0.001 (two-sided).

## Data Availability

The data (the second wave of the Midlife in the United States) in this study were provided by the Inter-University Consortium for Political and Social Research: https://www.icpsr.umich.edu/icpsrweb/ICPSR/studies/4652 (accessed on 6 May 2020).
